# ﻿*Lagerstroemiayangchunensis* (Lythraceae), a new species from Guangdong, China

**DOI:** 10.3897/phytokeys.255.138826

**Published:** 2025-04-08

**Authors:** Bao-Huan Wu, Xiu-Ting Liu, Zi-Yu Bao, Xiao-Yan Ma, Ying Chen, Guo-Di Chen, Xing Hu, Guo-Feng Liu, Se-Ping Dai

**Affiliations:** 1 Guangzhou Institute of Forestry and Landscape Architecture, Guangzhou 510405, China Guangzhou Institute of Forestry and Landscape Architecture Guangzhou China; 2 Guangzhou Horticultural Plant Germplasm Resource Nursery, Guangzhou 510405, China Guangzhou Horticultural Plant Germplasm resource Nursery Guangzhou China; 3 Guangzhou Collaborative Innovation Center on Science-Tech of Ecology and Landscape, Guangzhou 510405, China Guangzhou Collaborative Innovation Center on Science-Tech of Ecology and Landscape Guangzhou China; 4 Ting Jie Natural History Studio,Yangjiang 529500, China Ting Jie Natural History Studio Yangjiang China; 5 Yangjiang Municipal People’s Government Taiwan, Hong Kong and Macao Affairs Bureau, Yangjiang 529500, China Yangjiang Municipal People's Government Taiwan, Hong Kong and Macao Affairs Bureau Yangjiang China

**Keywords:** Crape myrtle, Flora, new species, Yangchun

## Abstract

A new species from southern China, *Lagerstroemiayangchunensis* B.H.Wu & G.D.Chen, **sp. nov.**, is described in the present paper. This species is morphologically similar to *L.duperreana* Pierre ex Gagnep., but can be distinguished by its coarse bark with vertical fissures, calyx lobes glabrous inside, and flowers with shorter pseudopedicels and petals with longer claws. Detailed morphological characteristics, habitat information, and comparisons with similar species are provided.

## ﻿Introduction

*Lagerstroemia* L. (Lythraceae) is a significant genus known for its ornamental value, notably for its vibrant summer blooms. Taxonomic revisions of *Lagerstroemia* have been extensive, commencing with Koehne’s monograph (Koehne 1883, 1903) and followed by subsequent revisions by Furtado and Srisuko (1969), which recognized 53 species. Subsequently, regional taxonomic updates (Lee and Lau 1983; Hewson 1990; Qin and Graham 2007; De Wilde and Duyfjes 2013, 2014, 2016; De Wilde et al. 2014) and additional species descriptions (Zhou et al. 2004; Gu et al. 2012, 2015; Deepu and Pandurangan 2017; Pham et al. 2017; De Wilde and Duyfjes 2019; Wu et al. 2023) have refined our understanding of the genus. Currently, the genus comprises ca. 52 species (Wu et al. 2023).

In recent years, we carried out several field surveys to gather germplasm resources of *Lagerstroemia* in China. In 2024, we found an unknown *Lagerstroemia* species with coarse and vertically fissured bark, a character uncommon in *Lagerstroemia* species of Guangdong. After extensive morphological comparisons and taxonomic analyses, we confirmed a new species which we formally describe herein.

## ﻿Materials and methods

Morphological characteristics were analyzed based on observations of the living plants in the field and dried specimens in herbaria. Measurements were conducted manually with rulers or using Digimizer version 4.6.0 (MedCalc Software, Mariakerke, Belgium). The voucher specimens were deposited in the herbarium of South China Botanical Garden (IBSC), South China Agricultural University (CANT), Sun Yat-Sen University (SYS) and China National Botanical Garden (CNBG).

## ﻿Taxonomic treatment

### ﻿ Lagerstroemia yangchunensis

Taxon classificationPlantaeMyrtalesLythraceae

B.H.Wu & G.D.Chen sp. nov.

A9319AC3-DAE0-53DF-A4C8-6A8A2DE9B553

urn:lsid:ipni.org:names:77359970-1

Fig. 1

#### Type.

China • Guangdong: Yangchun City, Chunwan Town, Xinglong Country, in forest, on sunny slope. 22.3327°N, 112.0097°E, 205 m a.s.l., 28 Jun 2024 (fl), *B.H. Wu Lg2024142* (holotype: IBSC!; isotypes: CANT!, CNBG!, SYS!).

#### Diagnosis.

*Lagerstroemiayangchunensis* is morphologically similar to *L.duperreana*, but distinguished by its coarse bark with vertical fissures, calyx lobes glabrous inside, and flowers with shorter pseudopedicels and petals with longer claws.

#### Description.

Trees to 15 m tall. Bark greyish brown, thick and coarse, vertically fissured; branchlets glabrous, terete. Leaves alternate, rarely subopposite; petiole 4–8 mm long, glabrous; leaf blade papery, margin entire, elliptic-oblong, oblong, rarely obovate, 10–14 × 3.5–5 cm, base cuneate to acute, apex acute, acuminate, rarely obtuse, adaxial surface green, glabrous, abaxial surface pale green, slightly pubescent along midvein, lateral veins brochidodromous, 10–15 on each side of midvein. Inflorescences paniculate, terminal or axillary; panicles 9–22 cm long, puberulous with whitish brown hairs. Pedicels 2–5 mm long, densely whitish tomentose; flower buds densely whitish tomentose, obovoid (excluding pseudopedicels), 7–9 mm long, pseudopedicel 2–3 mm long; flowering calyx tubes (excluding pseudopedicels) cup-shaped, 6–8 mm long, outside densely whitish tomentose, distinctly 12-ridged, inside glabrous, lobes 6, triangular to slightly acuminate, 2.5–4.3 × 2–3.5 mm, reflexed, epicalyx minute, pseudopedicel 2.5–5 mm long; petals 6, crumpled, ovate to broadly ovate, 15–20 mm long including 9–10 mm long claw; stamens 30–40, dimorphic, with 6 stamens longer (21–24 mm long), thicker and the lower half reddish purple in color, the remaining stamens shorter (10–13 mm long), thinner and white in color, filaments glabrous; ovary glabrous, styles 22–30 mm long, glabrous, stigmas small. Capsules oblong, smooth, 16–19 mm long, ca. 10 mm in diameter, loculicidally dehiscent, 5-valved. Seeds 7–10 mm long including wing.

**Figure 1.**
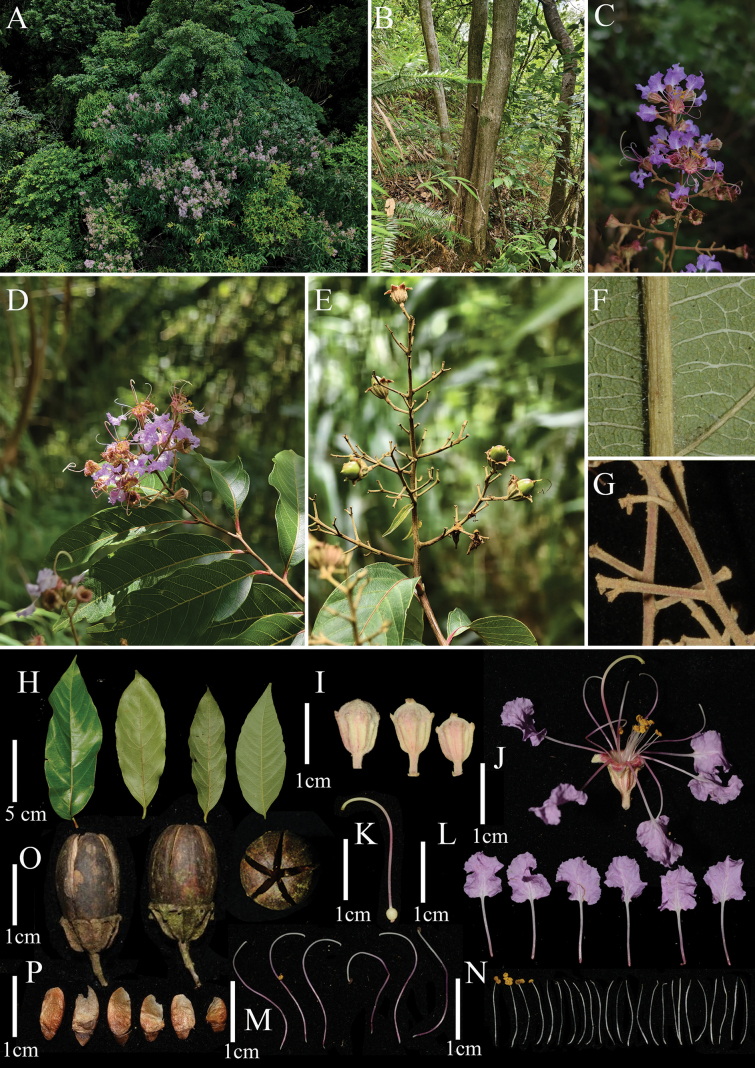
*Lagerstroemiayangchunensis***A** canopy view during flowering period **B** stems **C** flowering branchlet of the inflorescence **D** flowering branch **E** fruiting branch **F** part of the abaxial leaf surface **G** floral axes **H** leaves **I** flower buds **J** flower **K** gynoecium **L** petals **M** longer stamens **N** shorter stamens **O** capsules **P** seeds. Photographed by Guo-Di Chen and Bao-Huan Wu.

#### Phenology.

Flowering from June to July, fruiting after July.

#### Distribution and habitat.

*Lagerstroemiayangchunensis* is hitherto known from its type locality, Chunwan Town, Yangchun City of Guangdong; only 4 individuals were found in the population. It grows in forest on sunny slopes at ca. 200 m elevation.

#### Etymology.

The species epithet “yangchunensis” refers to Yangchun County, the locality where this species was discovered.

#### Vernacular name.

The proposed Chinese name for *Lagerstroemiayangchunensis* is 阳春紫薇 (yáng chūn zǐ wēi).

#### Discussion.

*Lagerstroemiayangchunensis* is morphologically similar to *L.duperreana*; however, it can be easily distinguished from the latter species by several characteristics, such as bark coarse and vertically fissured, calyx lobes glabrous inside and flowers with shorter pseudopedicels and petals with longer claws. A detailed comparison is summarized in Table 1.

**Table 1.** Morphological Comparison of *Lagerstroemiayangchunensis* and other similar species.

Characters	*L.yangchunensis*	*L.duperreana*	*L.speciosa*
Bark	greyish brown, thick, coarse, vertically fissured	light brown grey, thin, mottled and dimpled	light brown, black when old, coarsely vertically cracked and slightly flaking
Leaf shape	elliptic-oblong, oblong, rarely obovate	oblong, elliptic-oblong, sometimes obovate	elliptic, elliptic-oblong, or elliptic-ovate
Leaf size	10–14 cm long, 3.5–5 cm wide	8–12(–15) cm long, 3–5 (–10) cm wide	10–25 cm long, 6–12 cm wide
Leaf surface	adaxial surface glabrous, abaxial surface slightly pubescent along midvein	glabrous	glabrous
Number of lateral veins	10–15 on each side	8–10 on each side	(6–)8–14 on each side
Petiole length	4–8 mm	3–5 mm	6–15 mm
Panicle length	densely whitish tomentose	glabrous	glabrous
Flower buds	densely whitish tomentose, obovoid, 7–9 mm long, pseudopedicel 2–3 mm long	short densely whitish hairy, obovoid, ca. 6 mm long, pseudopedicel 6–12 mm long	short-farinose, subglobose, ca. 0.5 mm long, pseudopedicel 3–8 mm long
Calyx tube	6–8 mm long, distinctly 12-ridged	5–6 mm long, (indistinctly) 12-ridged	calyx tube 6–8 mm long, distinctly 12-ridged
Calyx lobe	2.5–4.3 mm long, glabrous within	ca. 3 mm long, densely whitish hairy in the upper part within	up to 8 mm long, glabrous within
Petal length	15–20 mm long, including 9–10 mm long claw	10–15 mm long, including ca. 5 mm long claw	ca. 20 mm long, including ca. 5 mm long claw

Having large inflorescences with flowers boasting vibrant purple colors, *Lagerstroemiayangchunensis* is a potential ornamental species for urban landscaping or *Lagerstroemia* breeding. However, its restricted distribution to the edge of a managed woodland in Chunwan Town poses significant threats to its survival. The small and isolated population, coupled with interference from human activities in its habitat, makes this species highly vulnerable to extinction. To ensure the species’ long-term viability and promote its sustainable development, urgent conservation measures, such as habitat protection, seed banking, ex situ cultivation, and public awareness campaigns, are necessary.

#### Additional specimen examined.

China • Guangdong Province: Yangchun City, Chunwan Town, 22.3327°N, 112.0097°E, 205 m a.s.l., 28 Jun 2024 (fl), *B.H. Wu Lg2024141*; *ibid.*, 28 June 2023, *B.H. Wu Lg2024143*, *Lg2024144*.

## Supplementary Material

XML Treatment for Lagerstroemia yangchunensis

## ﻿Additional information

### Author ORCIDs

Bao-Huan Wu https://orcid.org/0000-0002-0498-0603

Xiu-Ting Liu https://orcid.org/0009-0000-3383-5336

Zi-Yu Bao https://orcid.org/0009-0002-9768-2839

Xiao-Yan Ma https://orcid.org/0009-0007-7855-0984

Xing Hu https://orcid.org/0009-0001-8286-101X

### Data availability

All of the data that support the findings of this study are available in the main text.
